# Flogging tired horses: Who wants whipping and who would walk away if whipping horses were withheld?

**DOI:** 10.1371/journal.pone.0192843

**Published:** 2018-02-21

**Authors:** Paul D. McGreevy, Mark D. Griffiths, Frank R. Ascione, Bethany Wilson

**Affiliations:** 1 Sydney School of Veterinary Science, University of Sydney, Sydney, New South Wales, Australia; 2 International Gaming Research Unit, Psychology Department, Nottingham Trent University, Nottingham, United Kingdom; 3 Graduate School of Social Work, University of Denver, Denver, Colorado, United States of America; Gaziosmanpasa University, TURKEY

## Abstract

Recent studies have cast doubt on the effectiveness of whipping horses during races and this has led to questions concerning its continuing justification. Furthermore, it has been argued that whipping tired horses in racing is the most televised form of violence to animals. The present study used de-identified data from a recent independent Australian poll (n = 1,533) to characterise the 26% of respondents (113 females and 271 males) who support the whipping of racehorses and the 10% of racing enthusiasts in the sample (44 females and 63 males) who would stop watching races and betting on them if whipping were banned. Logistic regression models examining associations between age, gender, and income level of respondents demonstrated that those who support racehorse whipping are significantly more likely to be male. Among racing enthusiasts who would stop watching races and betting on them if whipping were banned, those in the lowest income bracket were over-represented. The more frequently respondents attended races or gambled on them, the more likely they were to agree that horses should be hit with a whip during the normal course of a race. These findings align with previous studies of violence among men and women but may also be attributed to male support of traditional gambling practices. Globally, racing organisations may consider the findings of the present study helpful in their deliberations on the merits of continuing the practice of whipping tired horses in the name of sport. The study might also provide important data for stakeholders who demand that it continues.

## Introduction

Ethical equitation demands that individuals cap the price horses pay for human glory [1,2]. Questions surround the ethics of any sport based on using animals, and particularly one that modifies an animal’s behaviour or pushes it to its physical limits via practices that cause the animal pain. Horseracing is steeped in tradition but is increasingly the source of various welfare concerns, such as the use of the whip and the physical dangers to horses involved in hurdle-racing and steeple-chasing. There is a pressing need for a scientific approach to training to be adopted in all equestrian pursuits, since this could obviate the need for whips, punishment, and the use of fear in escape learning [3].

The practice that is most obviously used on horses in the public eye is whipping them during a race. Increasingly, it has been justified as a means of improving jockey safety but, historically, one of the main defences for whip use in Thoroughbred racing was the belief that whipping made horses run faster [4]. Under the Australian Racing Board rules, only horses in contention of winning a race can be whipped, yet Evans and McGreevy [5] showed that, in a study of 15 races, 98% of horses studied were whipped. Horses, on average, achieved highest speeds in the sections of each race where no whip use was allowed, and increased whip use was seen most frequently when horses were fatigued. These results cast doubt on the effectiveness of whipping horses during races and its continuing justification.

Subsequent studies have examined the extent to which whip rules are policed [6], the differences in whip use between apprentice and senior jockeys [7], jockeys’ attitudes to whip use [8], and the impact of forehand and backhand whip use [9]. The padded whip has been regarded as the simple solution to concerns about whipping, but slow-motion videography has demonstrated that such whips strike horses with its unpadded section 64% of the time [10]. The same study showed that many whip-rule breaches are overlooked and others cannot be observed in any given focal horse, because competing horses obscure the viewer’s line of sight. These problems with surveillance by stewards may explain the recent revelations about discrepancies in whip-rule breaches that have raised questions about scrutiny (and thus, integrity) at country race meetings [11].

Jockeys argue that they need the whip for their own safety, because it can be used for steering and so can prevent horses colliding with one another or with fixed objects along the track. If the argument for the use of the whip in steering alone were valid, the whip would be of greatest benefit when used on the outside of bends on the course. McGreevy and Oddie [12] examined photographs of horses racing in New South Wales (NSW), where all racing is in a clockwise direction, and of horses racing in Victoria, where racing is counter-clockwise. Of 200 jockeys racing counter-clockwise, 91.5% held the whip in the right hand; of the 200 jockeys racing clockwise, 53.5% held the whip in the right hand. This indicates that placement of the whip appears to be primarily determined by the handedness of the jockey, not by the direction of the track. Given that more than half of NSW jockeys hold the whip in the inside hand, this particular study challenges the view that the whip is used for steering.

A desk study investigated associations between whip use and pre-race variables, including jockey experience, starting price, weight carried and barrier drawn [7]. If the whip is genuinely used in response to a given horse’s performance, there should be no consistent predictors of whip use. The study explored the influence of these variables on official whip counts for the race section 400m to 200m from the finish, and for the last section 200m from the finish. It concluded that apprentices whipped horses on average more than three times more than non-apprentice jockeys. These findings suggest that rider inexperience in Thoroughbred racing influences the number of whippings imposed on horses as they begin to tire.

A recent independent poll (commissioned, but not administered, by *RSPCA Australia*) explored the level of support for the whipping of racehorses among the Australian population, and the proportion of racing fans who would stop gambling if horses were no longer whipped. Three key findings from this study have been published. First, 74% responded that horses should not be hit with a whip during races. Second, 87% who watch or bet on racing would continue to do so if whipping were stopped. This finding is important, because the racing industry has long used the argument that serious punters want to see horses “ridden out” (i.e. ridden hard to the finish line) so that they can be satisfied that the horses they have backed have been given the best chance of winning. Third, among those watching and gambling on horseracing at least once a week or more, 90% said that they would continue to watch and bet even if whips were not used. The present study carried out secondary analysis on the de-identified data collected in the study commissioned by *RSPCA Australia* to characterise Australian respondents who support the whipping of racehorses and would stop gambling if whipping horses were to be banned.

## Method

### Participants and procedure

*RSPCA Australia* asked an independent research agency to conduct research into attitudes to whip use in racing. They received data on 1,533 individuals (731 males and 802 females) from around Australia on three questions as follows:

#### Question 1

“Thinking now about horseracing (including thoroughbred racing/gallops, and harness racing/trots), do you think horses should be hit with a whip in the normal course of a race?” Respondents could answer “Yes” or “No”.

#### Question 2

“In the last 12 months, how often have you watched and/or bet on a horserace?” Respondents could reply “Not at all”, “Once or twice (e.g. the Melbourne Cup)”, “At least once a month” or “At least once a week”.

After excluding those who had answered “Not at all” to this question, Question 3 was asked of respondents who had responded to Question 2 that they attended or bet on horseracing “Once or twice a year” or more frequently.

#### Question 3

“If the rules did not allow any horses to be hit with a whip (except in emergency/safety situations), would you continue to watch and/or bet on horseraces?” Respondents could answer “Yes” or “No”.

A nationally representative random sample was selected from the research agency’s current panel. The survey was 100% internet-based. It was not possible to give an accurate indication on the response rate due to the way respondents were invited to this agency’s surveys and because the agency uses invitations to re-activate members who have been inactive. The incidence rate of the survey was 82%, while everyone on the research agency’s current panel qualifies to take part, the agency applies quotas to ensure that their final data are representative of the Australian adult population (over 18 years of age). The completion rate was 98%, (i.e. 98% of respondents who started the survey completed it). As with all their surveys, the agency over-sampled by 5% to allow up to this amount to be removed during cleaning of the data. The three questions were posed as a stand-alone survey, i.e, not nestled in another context.

*RSPCA Australia*’s Chief Scientist (as data custodian) recently informed the authors of the availability of raw data from this online survey and provided access to the following information: (i) responses to three questions about horse whipping, (ii) age of respondents, (iii) household income level of respondents, and (iv) gender of respondents. The data collection for the present study was conducted with the approval of University of Sydney’s Human Research Ethics Committee (Approval number: 2017/442). The need for consent was waived by the ethics committee.

### Statistical analysis

Respondents’ answers to Questions 1 and 3 were analysed using the glm() function in the {stats} package of R (R Foundation for Statistical Computing, Vienna, Austria. URL https://www.R-project.org/). Odds ratios were calculated from the regression coefficients output by a logistic regression analysis, with confidence intervals provided by the profiling method offered by the {MASS} package. Responses to Question 2, as well as gender, age and income bracket, were considered as explanatory variables.

Additional, chi-squared statistics were calculated using the chisq.test () function of the {stats} package. For 2x2 tables, a Yates continuity correction was applied. For tables of larger dimensions, post-hoc pairwise chi-squared analyses were performed using the pairwiseNominalIndependence() function of the {rcompanion} package, with a Holm-Bonferroni correction for multiple comparisons.

## Results

### Question 1 (whipping horses)

When respondents were asked whether they thought horses should be hit with a whip in the normal course of a race, 1,149 respondents answered “No” (74.95%), and 384 answered “Yes” (25.05%).

#### Gender

Among the 802 female respondents, 689 answered “No” (85.91%), and the remainder answered “Yes” (14.09%). Among the 731 male respondents, 460 answered “No” (62.93%), while the remainder answered “Yes” (37.01%). Therefore, while both male and female respondents mostly reported that they did not think that horses should be whipped in the normal course of a race, males were more likely than females to think that they should (χ^2^ = 106.37, df = 1, p<0.001). This remained true once age, household income, and involvement with horseracing (as assessed by Question 2) had been accounted for [Odds ratio (OR) = 2.62; 95% confidence intervals (CI) 1.99–3.47].

#### Household income

The reported household income of those respondents who answered “Yes” or “No” to Question 1 appear in Table 1.

**Table 1:** The distribution of those who disagreed (n = 1,149) or agreed (n = 384) with whipping in the normal course of a horserace tabulated against the reported annual income of respondents.

Reported annual income (AUDK)	Number (and percentage) of remaining respondents who disagreed with whipping	Number (and percentage) of remaining respondents who agreed with whipping
<20	65 (73.86%)	23 (26.14%)
20–39	180 (82.57%)	38 (17.43%)
40–59	154 (71.96%)	60 (28.04%)
60–79	137 (75.69%)	44 (24.31%)
80–99	136 (71.58%)	54 (28.42%)
100–119	102 (73.91%)	36 (26.09%)
120–149	95 (66.90%)	47 (33.10%)
150–249	74 (62.18%)	45 (37.82%)
250+	19 (86.36%)	3 (13.64%)
NA	187 (84.62%)	34 (15.38%)

These results indicate that there is no simple relationship between household income and agreement with Question 1, and no income bracket was significantly different from the reference class of AUD20-39,999K in the logistic regression model.

### Question 2 (involvement)

Involvement with racing was assessed by responses to Question 2. Relationships between responses to Questions 1 and 2 are listed in Table 2.

**Table 2:** The distribution of those who agreed or disagreed with whipping in the normal course of a horserace among respondents who attended or bet on horseracing more frequently.

Frequency of attending or betting on races	Number (and percentage) of remaining respondents who disagreed with whipping	Number (and percentage) of remaining respondents who agreed with whipping
Not at all	621 (90.00%)	69 (10.00%)
1–2 annually	404 (76.95%)	121 (23.05%)
> monthly but < weekly	75 (47.17%)	84 (52.83%)
> weekly	49 (30.82%)	110 (69.18%)

Table 2 shows increasing agreement with whipping in the normal course of a horserace among respondents who attended or bet on horseracing more frequently (χ^2^ = 314.67, df = 3, p <0.001). Post-hoc pairwise testing resulted in p values less than 0.001 for all pairwise comparisons apart from “> monthly but < weekly” x “> weekly”, which resulted in p = 0.024.

When adjusted for age, gender, and household income by the logistic regression model, respondents who engaged with horseracing even “Once or twice annually” were more likely [OR = 2.64; CI 1.90–3.69] to agree with Question 1 than those who answered “Not at all” (for results of chi-square test, see above). More frequent attendees/gamblers were more likely still to agree with Question 1. Those involved at least monthly had an odds ratio of 7.30 (CI 4.83–11.09) and those involved at least weekly had an odds ratio of 15.91 (CI 10.37–24.76) when compared to non-attendees/non-gamblers (for results of chi-square test, see above).

### Question 3 (commitment to racing with whips)

Question 3 asked respondents if they would continue to watch/bet on horseracing if the rules were changed such that hitting horses with a whip was not allowed (except in emergency/safety situations). The 690 respondents who in response to Question 2 stated that they did not attend or bet on horseraces were not asked this question. Of the 843 remaining respondents, 736 reported that they would continue to watch/bet on horseracing if whipping were to be withheld except in emergencies (87.31%), and 107 responded that they would not (12.69%).

#### Gender

Question 3 was not asked to more than half of the original female respondents (415 of 802), because they indicated that they did not attend/bet on horseracing at all in their responses to Question 2. Of the remaining 387 women, 44 responded that they would not continue to watch/bet on horseracing if whipping were to be withheld except in emergencies (11.37%). This was a rate similar to male respondents, who answered that they would not continue to watch/bet (63 of 456; 13.82%; (χ^2^ = 0.92, df = 1, p = 0.337). Gender was not found to be of statistical significance in logistic regression modelling of responses to Question 3.

#### Household income

The reported household income of those respondents who answered “Yes” or “No” to Question 3 are listed in Table 3.

**Table 3:** The reported annual household income of racing fans (n = 843) who responded “Yes” or “No” to the likelihood of continuing to watch/attend races if whipping were to be withheld.

Reported income in AUDK	Number (and percentage) excluded by Question 2	Number (and percentage) of remaining respondents who answered “Yes”	Number (and percentage) of remaining respondents who answered “No”
<20	45 (51.14%)	34 (38.64%)	9 (10.23%)
20–39	126 (57.80%)	84 (38.53%)	8 (3.67%)
40–59	94 (43.93%)	108 (50.47%)	12 (5.61%)
60–79	80 (44.20%)	81 (44.75%)	20 (11.05%)
80–99	73 (38.42%)	103 (54.21%)	14 (7.37%)
100–119	57 (41.30%)	68 (49.28%)	13 (9.42%)
120–149	50 (35.21%)	77 (54.23%)	15 (10.56%)
150–249	32 (26.89%)	78 (65.55%)	9 (7.56%)
250+	6 (27.27%)	16 (72.73%)	0 (0.00%)
Not Answered	127 (57.47%)	87 (39.37%)	7 (3.17%)

Among those who answered Question 3, those with the lowest reported household incomes (less than AUD20K) were most likely to report that they would not continue to watch/bet on horseraces if whipping were to be withheld. Among the 43 respondents in this group, 9 answered “No” to Question 3 (20.93%), while 34 indicated that they would continue watching/betting if whipping were to be withheld (79.07%). Compared with these respondents, those whose household income exceeded 150K (the highest two income brackets combined), were significantly less likely [OR = 0.35; Ci 0.12–0.96, z = -1.986 p = 0.047] to say they would cease to watch or bet on horseracing if whipping were to be withheld, even after gender, age and involvement according to Question 2 were accounted for. For respondents in the middle-income brackets, the findings were more complex and responses to Question 3 were not significantly different from those in the lowest income bracket.

#### Involvement

Among Question 3 respondents, the levels of involvement as reported in response to Question 2 are listed in Table 4.

**Table 4:** The levels of involvement in racing among racing fans (n = 843) who responded “Yes” or “No” to the likelihood of continuing to watch/attend races if whipping were to be withheld.

Level of involvement	Number (and percentage) of remaining respondents to Question 3 who answered “Yes”	Number (and percentage) of remaining respondents to Question 3 who answered “No”
1–2 annually	463 (88.19%)	62 (11.81%)
> monthly but < weekly	130 (81.76%)	29 (18.24%)
> weekly	143 (89.94%)	16 (10.06%)

Unlike the respondents to Question 1, there is no simple relationship between degree of involvement and response among Question 3 respondents. Those who engage with horseracing at least monthly but less than weekly were most likely to report that they would not continue to watch horseracing under the scenario described by Question 3, but this difference did not reach statistical significance (χ^2^ = 5.78, df = 2, p = 0.056).

## Discussion

The main finding of the present study is that men are more supportive of whipping horses during horseraces than women. Even when age, household income, and involvement with horseracing are taken into account, males were more likely than females to have no problem with horses being whipped. This is, arguably, not surprising given that men are approximately eight times more likely to engage in violence generally than women [13] and that most animal crime offenders are male [14]. Using a scale for anthropomorphism designed by Albert and Bulcroft [15], studies by Duvall-Antonacopoulos and Pychyl [16] demonstrated that on a scale of increased anthropomorphism women scored higher than men. Consequently, if humans can relate to the possibility of whipping being painful, one would expect women to be less supportive of this activity. Another possible explanation is that awareness of horse welfare issues may differ between genders. This prospect is supported by Visser and Van Wijk-Jansen [17] who demonstrated that a subpopulation (cluster) of horse enthusiasts comprising 53% males was least interested in information on equine welfare while all clusters with a majority of females had much more interest in equine welfare. So, it is possible that, if they were horse enthusiasts, female respondents who attended horseraces or gambled on them, may be generally better informed about equine welfare than their male counterparts.

The more frequently respondents attended horseraces or gambled on them, the more likely they were to agree that horses should be hit with a whip during the normal course of a race. It cannot be presumed that most racing enthusiasts are aware of recent animal welfare concerns regarding the use of the whip in racing. It may be that the more exposed they are to whip use the more they see it as normal or the more they regard coercion as being needed to avoid jockeys throwing a race by not ensuring that the horses are pushed to their limits. It may also be the case that gamblers want to win money at any cost, even if it means that a horse is ill-treated.

In the context of horseracing, whipping can be considered violence only if the individual using the physical force intends to harm the animal. It is acknowledged that intent to cause harm is difficult to infer and this has led to the definition of animal abuse referring to “non-accidental” behaviour [18]. Allowing animals to be whipped in the name of entertainment and accepting the role of pain in this practice are at the core of the debate around this persistent issue. The horserace industry has steered clear of acknowledging that whipping might involve pain, insisting instead that horses recognise it simply as encouragement [19], but the industry offers no explanation for why this might be so. Pain or distress in non-human species is difficult to evaluate, but there is general acceptance that all mammals share the capacity to experience pain and this is recognised by legislation that governs the use of animals in research (i.e. that procedures and conditions that would cause pain and distress in humans will cause pain and distress in animals). Ironically, if a racehorse were whipped in the carpark outside the racetrack, the perpetrator would face charges under animal cruelty legislation outlawing any unnecessary, unjustifiable or unreasonable action that causes harm or injury, and could face significant fines or imprisonment. If whipping horses is considered a form of violence, the fact that it is aired during prime-time television and can be seen by minors may be of concern because it may normalise the meting out of physical punishment.

On-track whipping is not subject to animal-protection laws [20] but is regulated by a lower set of legal standards laid out in the Australian Rules of Racing. Horses move away from whip strikes, so it seems likely being whipped is aversive and that being whipped. Some industry veterinarians have even suggested that tired horses are running on adrenaline and therefore cannot feel any pain [19]. However, whether racing horses are adrenalinised to the point of being immune to pain as has been proposed [19] remains untested. How insensate horses can be ridden at all is puzzling, since we know that the application of aversive stimuli that are removed as soon as the animal responds as required (pressure-release or negative reinforcement) is the very foundation of good riding technique in all forms of equestrian sport. This logic would dictate that, in an analgesic state, horses running on adrenaline do not feel the bit in their mouths and so cannot be either decelerated or steered. Clearly, this is not the case, nor would it be safe to expect jockeys to ride such horses. Of course, the same line of reasoning would also mean that these horses could not feel the whip, and *that* argument would render whipping useless. Those who believe horses should be whipped may have aligned with this view or may believe that horses that are underperforming deserve physical punishment–a rationale sometimes delivered by those parents who physically abuse their children or by abusive adult partners (most often men).

Among those who answered Question 3, those with the lowest reported household incomes were most likely to discontinue watching/betting on horseraces if whipping were to be withheld. In contrast, those in the highest two income brackets (combined) were significantly less likely to do so. Arguably those in the highest two income brackets have a very different motivation to gamble on horseracing compared to those on the lowest incomes. It may be more likely that wealthy individuals can afford to lose money and attend races more for social purposes or in the context of corporate events where the gambling is more incidental or for ego-enhancement purposes and winning money is secondary [21]. For those on low incomes, the motivation for gambling may be more likely to be a financial one, where winning money is paramount and where such gamblers believe that winning money will solve their financial problems [22]. Here, the low-income gamblers’ emotional pain of losing is likely to be far more salient to the individual than the physical pain caused to horses that they have bet on. Ultimately, losing money on horserace gambling will have a much more devastating psychological effect on low-income individuals than on high-income individuals, so low-income groups are much more likely to be focused on the outcome of the race and who the winner is than on the way the horse and jockey won the race. A low-income gambler who wins money on a horse that has been excessively whipped is unlikely to care much about the welfare of the horse.

If people would stop watching races and betting on them in the absence of whip use, this is most plausibly explained by their belief that races are not fair, and somehow lack integrity, if horses are not seen to be “ridden out” on their merits (i.e. are not whipped). This view is not supported by the persistence of Thoroughbred races (and gambling on them) in Norway where whip use has been forbidden for more than 30 years. It also runs counter to recent evidence that whip use is not associated with improved placings in Thoroughbred races [5]. Furthermore, in harness racing, a recent study explored relationships with two rule changes that moderated whip use. The first (October 2010) moderated whip action so that horses were struck with less force. A comparison of the frequency of fast, medium, and slow winning times before and after the rule change showed that there were significantly more fast and medium winning times in the later period. The second (more modest) change (2016) was associated with no change in race times [23]. Given that whip use has been associated with shortened strides that are unlikely to improve performance [24] and falls that compromise jockey safety [25, 26], it is unsurprising that its place in sustainable models of horseracing has been questioned [27].

The current study has demonstrated that among those who regularly watch races and bet on them, those who would stop watching or gambling on horseraces if whips were banned were more likely to be male. It is possible that these may be gamblers who are seeking to quit and are happy to embrace the influence of an externally imposed change on their preferred choice of gambling, a change they can argue has changed the rules untenably.

## Conclusions

This study has revealed that those who support racehorse whipping are significantly more likely to be male. The more frequently respondents attended races or gambled on them, the more likely they were to agree that horses should be hit with a whip during the normal course of a race. These findings align with previous studies of violence among men and women but may also be attributed to male support of traditional gambling practices. Among racing enthusiasts who would stop watching races and betting on them if whipping were banned, those in the lowest income bracket were over-represented. Globally, racing organisations may consider the findings of the present study helpful in their deliberations on the merits of continuing the practice of whipping tired horses in the name of sport.
